# Abducens Nerve Palsy as a Complication of Herpes Zoster Ophthalmicus: A Case Report

**DOI:** 10.7759/cureus.22920

**Published:** 2022-03-07

**Authors:** Anas Al-sadi, Mohammed Abdulgayoom, Israa Jawarneh, Akram Al-warqi

**Affiliations:** 1 Internal Medicine, Hamad General Hospital, Doha, QAT; 2 Internal Medicine, King Abdullah University Hospital, Irbid, JOR; 3 Radiology, Hamad General Hospital, Doha, QAT

**Keywords:** abducens nerve palsy, sixth cranial nerve palsy, ophthalmoplegia, herpes zoster ophthalmicus, herpes zoster

## Abstract

Herpes zoster (shingles) is a common viral infection that results from the reactivation of varicella-zoster virus (VZV), which remains dormant in sensory ganglia after initial infection. The usual presentation is radicular pain followed by eruption of vesicular rash. herpes zoster ophthalmicus (HZO) is defined as the involvement of ophthalmic division (V1) of the trigeminal nerve (V).

Extraocular muscle paralysis is a rare complication of HZO. Here, we report a case of HZO that developed abducens nerve (VI) palsy and secondary raised intra-ocular pressure.

## Introduction

Herpes zoster (shingles) results from reactivation of the dormant varicella-zoster virus (VZV), characterized by unilateral radicular pain and a vesicular eruption in a dermatomal distribution [1]. VZV most often affect sensory neurons, but motor neuron can be involved in rare cases [1,2].

Herpes zoster ophthalmicus (HZO) is defined as herpes zoster involvement of the ophthalmic division of the fifth cranial nerve (V1). Ophthalmoplegia can complicate it in rare cases [3], with the oculomotor nerve (III) being the most frequently involved, followed by the abducens (VI), then the trochlear (IV) nerve [2]. Ophthalmoplegia usually appears 2-4 weeks after the rash but sometimes co-occur together. The prognosis for recovery is good, with significant improvement typically seen within two months and complete or near resolution within 18 months [4].

The sixth cranial nerve (abducens nerve) innervates the ipsilateral lateral rectus muscle, responsible for eye abduction. Abducens nerve palsy presents with binocular horizontal diplopia, sometimes with head-turn to minimize diplopia. Most cases are acquired. The most common cause is microvascular ischemia related to diabetes and hypertension. Other less common causes include neoplasm, multiple sclerosis, stroke, sarcoidosis, and elevated intracranial pressure (false localizing sign) [5]. HZO as a cause of abducens nerve palsy is reported in a few cases.

Here, we describe a case of an immunocompetent young patient who developed unilateral abducens nerve palsy as a complication of HZO.

## Case presentation

Our patient is a 49-year-old male with well-controlled type 2 diabetes and a history of right-shoulder osteochondroma treated with a local steroid injection. Metformin/vildagliptin is his only home medication. Presented with acute pain across the right ophthalmic nerve (V1) distribution for five days and had a documented fever at 38.9 degrees Celsius.

**Figure 1:**
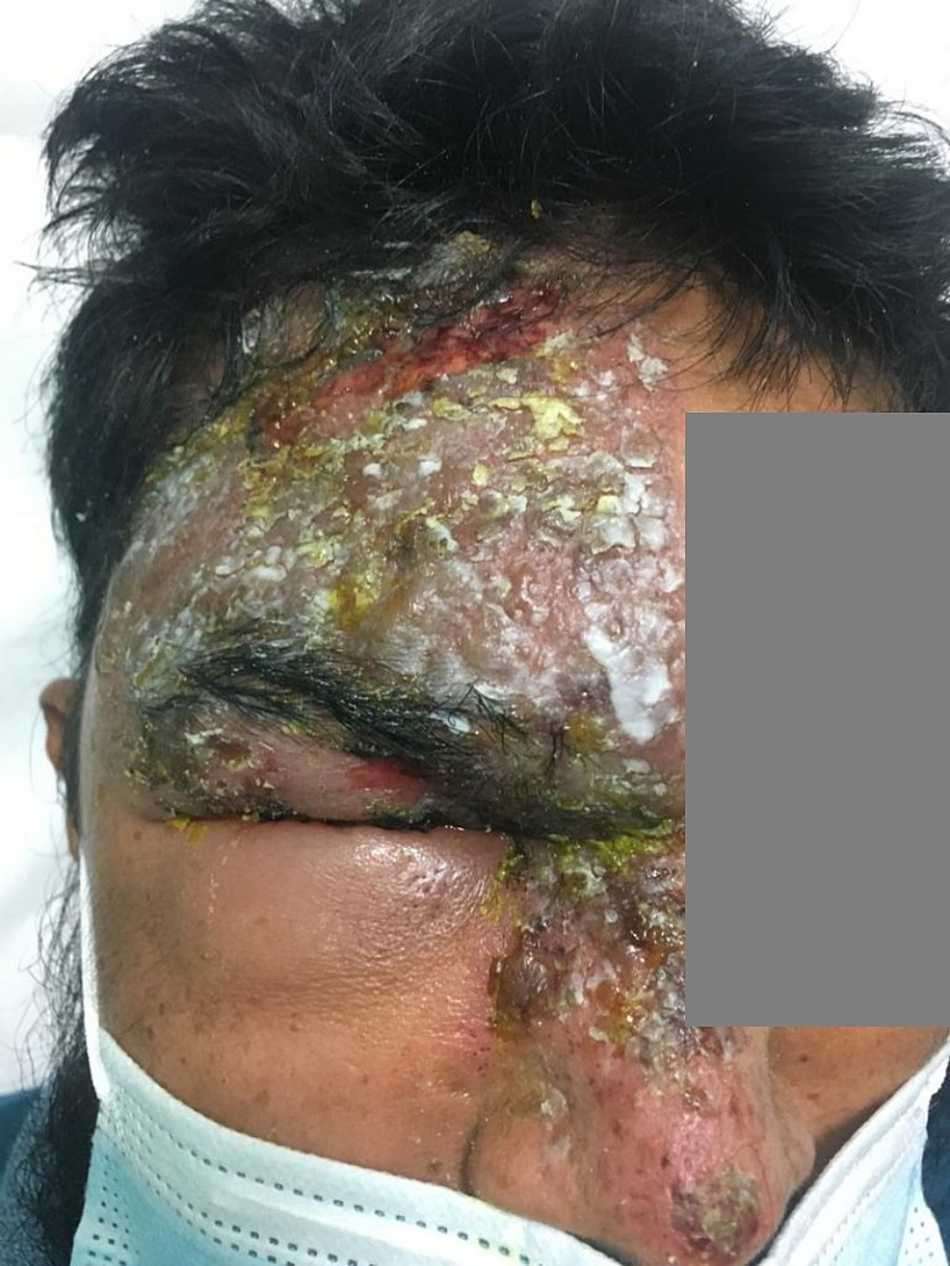
Extensive vesicular skin rash over the right V1 distribution with eyelid edema and positive Hutchinson's sign.

He developed extensive vesicular skin rash with excoriation over the right V1 distribution three days later with worsening eyelid edema and positive Hitchnson's sign (Figure 1). He was diagnosed with HZO and treated with oral acyclovir, pain relievers, and a topical eye steroid at the emergency room and he was discharged home.

However, his condition did not improve, and his eyelid edema deteriorated to the point that he could not open his right eye even with help, so he was admitted to the hospital two days later. At this point, ophthalmologic testing was limited due to considerable eyelid edema.

Investigations were remarkable for positive varicella-zoster antibody IgG and negative for IgM. CBC and CMP were unremarkable and HIV was negative. HbA1c was 6.6%.

**Figure 2:**
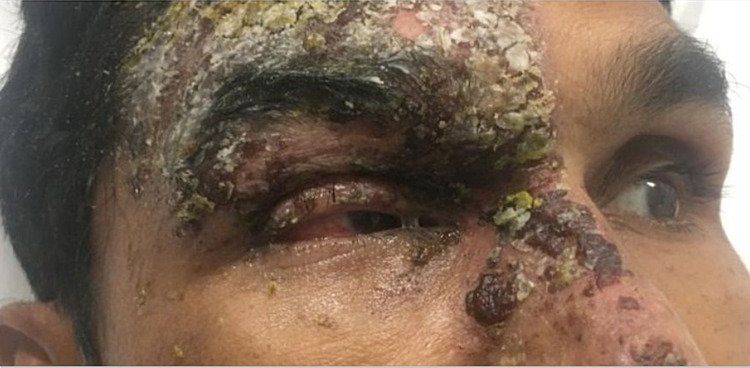
Improvement of eyelid edema with intact extraocular muscle movement toward the left side.

**Figure 3:**
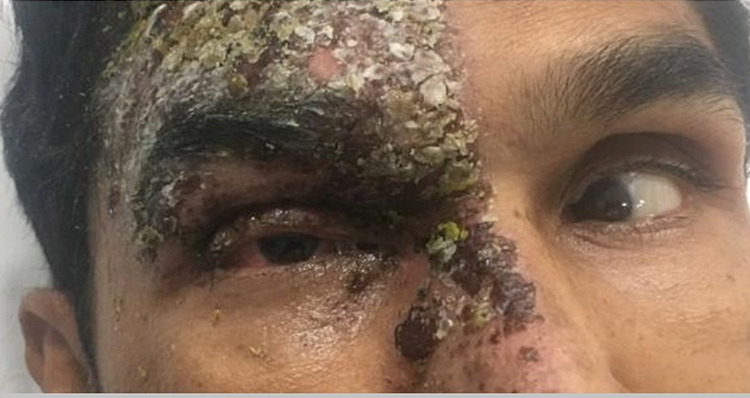
Right eye was unable to abduct associated with horizontal diplopia, finding consistent with a diagnosis of abducens nerve palsy.

**Figure 4:**
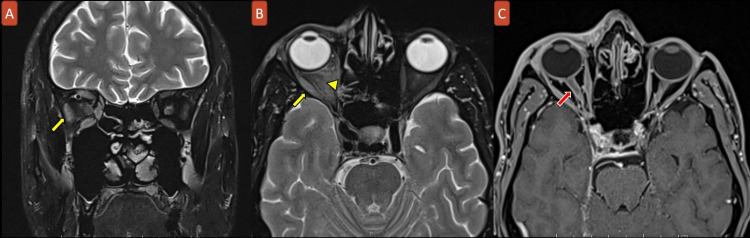
MRI brain with fat saturation in coronal T2WI (A) and axial T2WI (B). Axial T1WI with contrast (C) showing mild diffuse enlargement of the extraocular muscles in the right orbit with increased enhancement (yellow arrows) in addition to diffuse optic nerve sheath enhancement in the right orbit (red arrow) with mild retrobulbar orbital fat haziness (yellow arrowhead) indicating orbital myositis and preoptic neuritis.

After two days of IV Acyclovir, edema and rash began to improve, and he became able to open his right eye. Examination showed limited right eye abduction associated with horizontal diplopia consistent with right abducens nerve palsy (Figures 2, 3). A slit-lamp study revealed a small dendritic ulcer on the cornea, as well as increased intraocular pressure of 25 mmHg. An MRI head was performed to rule out other causes for abducens nerve palsy and showed mild diffuse extraocular muscle enlargement and enhancement with optic nerve sheath enhancement in the right orbit (Figure 4).

After he improved, we discharged the patient on oral valacyclovir, topical prednisolone, and timolol eye drop. The intraocular pressure and nerve palsy improved with follow-up in an ophthalmology clinic one month later, but the patient returned to his home country and lost to follow-up.

## Discussion

Infection with the varicella-zoster virus (VZV) results in two different illnesses. Varicella (chickenpox) is caused by primary infection with VZV and is characterized by vesicular lesions in various stages of development on the face, trunk, and extremities. Herpes zoster, more often referred to as shingles, is caused by the reactivation of dormant VZV infection inside the sensory ganglia [6].

HZO, a potentially blinding disorder, is described as herpes zoster involvement of the fifth cranial nerve's ophthalmic division. In several surveys, the incidence of HZO complicating herpes zoster ranged between 8% and 20%. If antiviral medication is not administered, approximately 50% of individuals with HZO will have direct ocular involvement, like conjunctivitis, uveitis, episcleritis, keratitis, and acute retinal necrosis [7,8].

Peripheral motor neuropathy is an uncommon but well-established consequence. It occurs in roughly 3% of patients with herpes zoster and is caused by VZV spreading from the dorsal root ganglia to the anterior root/horn of the spinal cord [9,10]. On the other hand, only a few papers have focused on HZO-associated ocular motor paralysis [11].

HZO has been reported to cause extraocular muscle palsies in the third, fourth, and sixth cranial nerves. The third nerve is the most frequently encountered, while the fourth is the least [12].

Extraocular muscle palsies often develop 2-4 weeks after the rash but can occur concurrently or more than four weeks afterward. Our patient was found to have palsy of the sixth nerve one week after developing the vesicular rash [2]. The pathophysiology of ophthalmoplegia is debatable, and different hypotheses have been suggested.

Denny-Brown et al. [13] reported that motor neuritis occurred independently of ganglion inflammation. Edgerton and Godtfredsen et al. [14,8] hypothesized that the second, third, fourth, and sixth nerves were involved as a result of continuous intracavernous radiculomeningitis. Kreibig et al. [1] proposed that perivasculitis-myositis rather than a neurological cause caused extraocular palsies.

From this, we may conclude that the pathophysiology of ophthalmoplegia may be attributable to one of the following: direct cytopathic impact of the virus on the brain tissue it infects, central nervous system's immunological reaction to the infection, or attributes it to a virus-induced occlusive vasculitis [1].

Antiviral therapy aims to promote faster healing of skin lesions, alleviate the degree and length of pain associated with acute neuritis, and potentially minimize the occurrence or severity of persistent pain, also known as postherpetic neuralgia [12].

Antiviral therapy and steroid have been suggested for paralytic lesions caused by herpes zoster; however, the benefits of a particular treatment are unknown because it tends to resolve spontaneously [2].

In general, it has been noted that the prognosis for spontaneous recovery of extraocular muscle function is favorable [15].

## Conclusions

Ophthalmoplegia in HZO may require a thorough workup including imaging to rule out other possible causes of cranial nerve palsy. The underlying pathophysiology of abducens nerve palsy in patients with HZO is not well established but could be due to myositis or neuritis, among other possible causes. The benefit of antiviral medications and corticosteroids in treating oculomotor palsy remains debatable, especially in light of reports of spontaneous recovery.
